# Hyperspectral retinal imaging biomarkers of ocular and systemic diseases

**DOI:** 10.1038/s41433-024-03135-9

**Published:** 2024-05-22

**Authors:** Abera Saeed, Xavier Hadoux, Peter van Wijngaarden

**Affiliations:** 1 Centre for Eye Research Australia, Royal Victorian Eye and Ear Hospital, Melbourne, 3002 VIC Australia; 2 Ophthalmology, Department of Surgery, University of Melbourne, Melbourne, 3002 VIC Australia

**Keywords:** Predictive markers, Brain injuries

## Abstract

Hyperspectral imaging is a frontier in the field of medical imaging technology. It enables the simultaneous collection of spectroscopic and spatial data. Structural and physiological information encoded in these data can be used to identify and localise typically elusive biomarkers. Studies of retinal hyperspectral imaging have provided novel insights into disease pathophysiology and new ways of non-invasive diagnosis and monitoring of retinal and systemic diseases. This review provides a concise overview of recent advances in retinal hyperspectral imaging.

## Abstract (zh)

高光谱成像是医学影像学技术领域的前沿。它可同时收集光谱和空间数据。编码于这些数据中的结构和生理信息可用于识别和定位典型的难以捕捉的生物标记物。视网膜高光谱成像研究为疾病的病理生理学提供了新的见解, 为视网膜和全身疾病的非侵入性诊断和监测提供了新方法。本文简要阐述了视网膜高光谱成像的最新进展。

## Introduction

Our perception of the visual world is intricately tied to the unique and specific interaction of light with matter. This interaction varies as a function of the composition of matter and the physical properties of the illuminating light. In the laboratory, interrogation of this interaction to gain insights into the composition of an object is the basis of spectroscopy. At a simpler level, conventional photography provides insights into the retinal structure through the interaction of broadband light with the retina that is detected by a three channel (red, green, and blue) sensor, that emulates human cone photoreceptor sensitivities and can thus recapitulate what is seen by the eye. Whilst clinically useful, this imaging method is inherently constrained. More detailed information about the structure of the retina can be acquired by interrogating the wavelength-specific interaction of light with the tissue. This is intuitive for most clinicians—a green (*red-free*) filter is commonly used for slit-lamp biomicroscopy to highlight retinal haemorrhages, due in part to the fact that haemoglobin in blood is a strong absorber of green light. Hyperspectral imaging is an extension of this approach, and it involves the acquisition of a series of images of the retina at different wavelengths of light. The high-dimensional information that is acquired in this manner has the potential to transform biomarker detection for a range of eye and systemic diseases and to advance understanding of pathophysiology.

## Principles of hyperspectral imaging

Hyperspectral imaging has its origins in geospatial sciences [1] but has evolved over several decades, with expanding applications in art conservation [2], food quality and safety control [3, 4], pharmaceuticals [5], agriculture [6], forensics [7, 8] and medicine [9, 10].

**Fig. 1:**
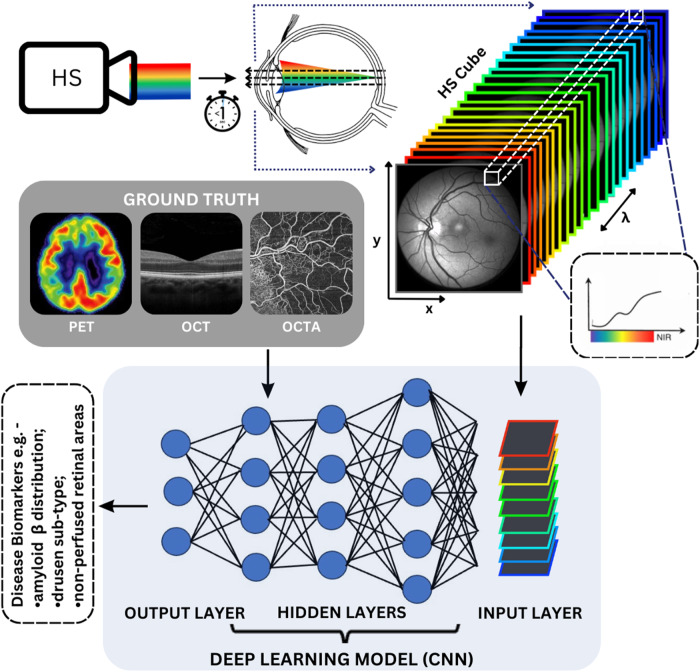
Schematic of hyperspectral imaging. A hyperspectral imaging camera (labelled HS) uses narrow bandwidth tuneable light source to illuminate the retina typically in less than one second. The reflected light from the retina is then collected by an image sensor. There are various modes of acquiring a hyperspectral image, but typically different frames are obtained by scanning the source wavelengths to generate a data cube (HS Cube). Each image has both spatial and spectral information and each pixel has a corresponding spectral signature. Images are analysed using deep learning image analysis methods. A convolutional neural network (CNN) is illustrated here. A CNN model is composed of input, hidden and output layers. Hidden layers are fully connected and consist of multiple stacks of convolution, pooling and activation, enabling automated feature extraction, classification and regression. Training a CNN model requires accurate ground-truth data, depicted here as positron emission tomography (PET), optical coherence tomography (OCT) and optical coherence tomography- angiography (OCTA) scans for illustrative purposes. Outputs of this model include disease biomarkers.

Hyperspectral imaging is like conventional photography, however instead of using a single broadband (*white*) light flash, a series of images is captured across a continuous range of discrete wavelengths of interest. These images are then stacked to yield a three-dimensional data set called a *hypercube*, which is comprised of two spatial dimensions (x and y) and one spectral dimension (λ) (Fig. 1) [11]. Each location, or pixel in the hypercube has its own spectral signature (reflectance as a function of wavelength) indicative of its composition [12] and can be further interrogated to identify the individual constituents (referred to as endmembers).

### Modes of acquisition

A variety of hyperspectral imaging methods have been developed [11, 13–16]. As imaging sensors typically collect information in two dimensions, and as hyperspectral imaging data is three-dimensional, two dimensions are captured simultaneously, while the third dimension is scanned through. The *spatial-scanning* method, often referred to as the *push-broom scanner*, involves scanning point by point or line by line in the spatial dimension in a ‘sweeping’ motion. This often results in a low frame rate and extended acquisition times [14]. The *spectral-scanning* method preserves spatial resolution by acquiring a two-dimensional image at a specific wavelength and then scans along various wavelengths to obtain the whole hyperspectral image [16]. The main disadvantage of these methods is they require visual fixation and image registration to reduce motion artefacts [17]. The *snapshot method*, on the other hand, achieves a very high frame rate without scanning by acquiring the image through a pixel-sized bandpass filter integrated with the image sensor [11, 16]. Although it offers a swift frame rate, pixel convolution is necessary, leading to lower spatial resolution. Multispectral imaging is a variant of hyperspectral imaging using multiple broader band wavelengths that are fewer in number (typically between three to ten) than are used in true hyperspectral imaging (ten to hundreds of narrow wavelength bands) [11, 13].

### Hyperspectral data processing

Hyperspectral image data cubes are complex and require processing prior to analysis [11, 13]. Each pixel in a hyperspectral image consists of a mixture of reflectance spectra of endmembers [13, 18]. Spectral unmixing is a commonly used analysis technique. Unmixing aims to isolate the individual endmembers present at a given location, and to quantify the abundance of each endmember in each pixel at that location. In the past, mathematical models that account for unmixing have been constructed and used with some success [16, 17]. More recently, approaches utilising artificial intelligence have been popular because of their ability to process and analyse large amounts of highly complex data [16, 19, 20]. A comprehensive discussion of hyperspectral imaging data analysis techniques is beyond the scope of this review and can be found elsewhere [21, 22].

## Clinical applications of hyperspectral imaging

Recent advances in hyperspectral cameras, image analysis methods, and computational power have catalysed the application of hyperspectral imaging in a variety of fields of medicine, including ophthalmology [21]. Owing to physiological constraints, including the phototoxicity and limited ocular penetration of ultraviolet light, most applications of hyperspectral imaging in ophthalmology are limited to the visible and near infrared spectrum of light. Exploratory studies of hyperspectral and multispectral imaging have been reported for Alzheimer’s disease (AD) [20, 23, 24], age-related macular degeneration (AMD) [25–27], diabetic retinopathy (DR) [28], retinal vein occlusion (RVO) [15, 29] and glaucoma [30, 31]. Herein, we provide a summary of some these studies with a particular focus on applications for retinal oximetry, AMD and AD due to the considerable attention they have garnered in this field.

### Retinal vascular diseases

Retinal hypoxia is a key component of numerous ocular diseases including diabetic retinopathy [32], retinal vein occlusion [15, 29], and some forms of glaucoma [30, 31]. Whilst fluorescein angiography (FA) and OCT-angiography (OCTA) are used as surrogate markers of hypoxia, high resolution and non-invasive retinal oximetry methods have the potential to significantly improve the detection, prognostication and management of these diseases [1].

The characteristic absorption spectra of oxy- and deoxy-haemoglobin serve as the basis of pulse oximetry and non-contact retinal oximetry. Oxymap T1 (Oxymap, Reykjavík, Iceland), the reference device for retinal oximetry, uses dual wavelength imaging to estimate oxygen saturation values for higher order vessels [32, 33]. Hyperspectral imaging has several potential advantages over dual wavelength oximetry. The use of many spectral channels can facilitate noise reduction and improved signal detection [30]. This can enable the development of more complex oximetry models and potentially enable pixel-level oximetry across the image field [17]. Studies of a range of retinal diseases indicate that hyperspectral imaging may enable high resolution retinal oximetry to improve our understanding of disease pathophysiology [15, 31, 34–38].

A variety of spectral imaging methods have been used to accurately measure oxygen saturation values in the larger retinal vessels in humans [15, 39]. However, achieving oximetric measurements of the retinal tissue, away from the larger vessels, is more difficult due to signal-to-noise challenges. The feasibility of retinal tissue oximetry has been demonstrated using multispectral imaging, coupled with sophisticated data processing methods, for human retina [15, 30, 40] as well as for the optic nerve head of animal models [41, 42]. Unfortunately, traditional multispectral imaging methods are limited by lengthy acquisition times, requiring prolonged visual fixation and image registration to reduce motion artefacts. Snapshot hyperspectral systems acquire spatial and spectral information simultaneously, and due to the lack of scanning, allow for visualization of dynamic processes free of motion artefact. Dwight et al. used the Image Mapping Spectrometer (IMS), a snapshot hyperspectral imaging camera with high spatial sampling density (350 × 350) and 40 spectral channels to measure absolute oxygen saturation values without the need of calibration in patients with a range of retinal diseases (including exudative and non-exudative AMD, glaucoma, retinitis pigmentosa and uveitis) [17]. As the spectral range of this system spans from green to near-infrared wavelengths, it is possible to visualise the retinal and choroidal circulations. Accordingly, it may be possible to derive choroidal and retinal oximetry measurements in future using a similar approach. As little is known about human choroidal oxygen saturation in disease, this advance may enable important new pathophysiological insights. Furthermore, the study proposed that improved detector array performance may enable the generation of video rate oximetry maps of retinal vasculature which may offer useful information about oxygen dynamics [17]. The ability to compute retinal oximetry maps with high spatial and temporal resolution has significant clinical potential however further clinical studies are required to move this technology from proof-of-concept stage [43].

A study by Li et al. [28] showed that multispectral imaging was comparable to fluorescein angiography (FA) and superior to conventional retinal photography for grading diabetic retinopathy. Key retinopathy features, including microaneurysms and intraretinal microvascular abnormalities were more readily detected with multispectral imaging than with conventional photography [44]. A subsequent study further explored hyperspectral imaging as an alternative to FA [40]. FA remains a gold standard for evaluating retinal vasculature however it is an invasive, lengthy procedure with associated risks. Dwight et al. utilised a *snapshot* hyperspectral camera (IMS) and a novel unmixing method to measure oxyhaemoglobin abundance (HbO_2_) in the retina analogous to FA [40]. They generated oxyhaemoglobin maps that correlated with areas of hypo- and hyperfluorescence in the venous phase of FA images however some discrepancies did exist. While the use of hyperspectral imaging may have promise as a non-invasive analogue of FA, it cannot recapitulate dynamic features such as leakage, pooling and staining. The operational constraints of this method remain to be seen, such as the impact of intraretinal haemorrhage or pigment on oximetry measurements, or the length of time required for post-image processing [40]. It is likely that insights gained from hyperspectral imaging oximetry may serve as useful adjuncts to those from FA and OCTA.

### Age-related macular degeneration (AMD)

Improved biomarkers for the detection of AMD and its prognostication are important to facilitate clinical trials of emerging therapies and, ultimately, to identify those who stand to benefit from them [27]. Exploratory studies of hyperspectral imaging and visible light spectral-domain optical coherence tomography have shown promise for AMD biomarker detection [26, 45, 46].

Drusen are a hallmark feature of AMD and current conventional in vivo imaging techniques have a limited capacity to distinguish between drusen subtypes based on their composition. The use of *snapshot* hyperspectral imaging and non-negative matrix factorization has been used to identify characteristic spectral profiles for drusen that are distinct from drusen-free regions of the macula in a small study of people with AMD [25]. This study also reported the first in vivo demonstration of absorbance peaks for the two main carotenoids of macular pigment, lutein and zeaxanthin [25]. In a subsequent study, computed tomography *snapshot* imaging spectrometry was used to quantify macular pigment in a group of healthy eyes in vivo [1, 47].

Changes in the retinal pigment epithelium (RPE) and accompanying abnormalities in retinal autofluorescence (AF) signals are strong indicators for AMD and its progression [26, 48]. Lipofuscin and melanofuscin are two key autofluorophores that accumulate within RPE in AMD. AF attributable these molecules have a broad emission spectrum which represents a sum of multiple constituents [49]. Ex vivo autofluorescence hyperspectral imaging of normal RPE flatmounts has identified distinct spectral signatures for lipofuscin and melanofuscin [26, 45]. In subsequent studies of human retinal flatmounts with AMD, a spectral signature for drusen and sub-RPE (basal laminar and basal linear) deposits was identified at an emission peak of 510 nm [45]. Drusen and sub-RPE deposits could not be spectrally distinguished in this study. This initial effort to decompose retinal AF emission spectra into their separate constituents using hyperspectral imaging is a step towards molecular identification of these fluorophores in vivo.

The use of visible light OCT for hyperspectral imaging is a promising advance that may enable acquisition of axially-resolved retinal spectral data. The approach has been used to assess melanin levels in the RPE of albino and pigmented rodents [46]. This is pertinent to AMD pathophysiology as melanin is considered to guard against cell damage in the disease [11, 46]. While the method was effective for measuring melanin levels in rats, it was less effective in pigmented mice which have melanin levels closer to those in humans. Advances in this technology may enable in vivo quantitative melanin imaging in human RPE and other retinal layer-based spectral analyses in future.

High-fidelity imaging retinal densitometry, is a novel multispectral imaging method, which aims to measure visual pigment densities in AMD [50]. Imaging employs LED arrays and infrared pupil tracking, to map visual pigment densities. This method has been shown to discern photoreceptor cell types and track pigment synthesis rates, which are notably reduced in people with intermediate AMD compared to those without, despite comparable photoreceptor densities. These findings may serve as the basis of an early biomarker of AMD [50].

### Alzheimer’s disease

Alzheimer’s disease (AD) is the leading cause of dementia and the recent development of disease-modifying therapies, coupled with a limited availability of reliable, convenient, and affordable tests for the disease has underscored the need for improved biomarkers. The retina is a developmental extension of the forebrain and several of the cardinal pathological changes that occur in the brain, such as the accumulation of amyloid β and phosphorylated tau, also occur in the retina [51–53]. Accordingly, numerous studies have examined the potential of retinal imaging biomarkers of AD.

One of the first investigations of hyperspectral imaging in AD identified a spectral signature for aggregated amyloid β in a neuronal cell line in culture [54]. A similar spectral signature was found with ex vivo hyperspectral microscopy of retinal flatmounts of transgenic AD model mice that was distinct from that of age-matched wild-type control mice [23, 54, 55]. Furthermore, the spectral signature for amyloid β was also identified in human brain and retinal samples from people with histopathologically-confirmed AD and this signature was not found in the tissues of age-matched AD-free individuals. In vivo topical hyperspectral retinal endoscopy was then demonstrated to detect a spectral signature for amyloid β in AD-model mice [54, 56]. Hadoux and colleagues subsequently reported the use of hyperspectral retinal imaging to distinguish people with mild cognitive impairment or early AD, who were positive for amyloid β on brain positron emission tomography (PET) imaging, from age-matched controls who were PET negative for amyloid β. A subsequent study reported prediction of amyloid β PET status from hyperspectral imaging measures of retinal texture and vascular parameters (tortuosity and diameter) [57]. A third study showed that retinal spectral differences between people with clinically defined AD and controls was greatest for subjects with moderate and minimal reductions cognitive test scores (Mini-mental State Examination scores ≥22), suggesting that hyperspectral retinal imaging may have most value in early-stage disease.

More recent laboratory studies of hyperspectral microscopy of retinal tissue sections has identified distinct spectral signatures for phosphorylated tau (pS396-tau) and amyloid β which, when analysed with deep learning algorithms, could be used to recapitulate immunohistochemical staining patterns for p-tau and amyloid β with a high degree of accuracy [53]. Further studies are required to ascertain whether a spectral signature for phosphorylated tau can be detected in vivo and larger clinical studies are needed to determine the clinical utility of retinal biomarkers of AD. In any event, these studies provide proof-of-principle of the capacity of hyperspectral imaging for the detection of low abundance, disease-relevant molecules for the development of advanced imaging biomarkers.

## Challenges and future directions

Hyperspectral imaging has great potential for biomarker discovery for a range of retinal and systemic diseases. Whilst the application of this technology for retinal imaging is still nascent, early studies have shown promise for improved disease detection and insights into disease pathogenesis. Challenges exist for the translation of this imaging method into clinical practice. At present, there are few commercially available hyperspectral cameras and most clinical studies have been proof-of-concept. Sufficiently powered cross-sectional studies with gold standard ground truth comparators are required to evaluative putative diagnostic biomarkers. Similarly, longitudinal studies are needed to identify biomarkers for disease progression and prognostication.

Deep learning has emerged as a valuable tool for hyperspectral imaging data analysis. The novel findings that have been provided by the use deep learning algorithms for the analysis of conventional retinal photographs, a highly constrained input data source, hint at the enormous potential of deep learning for hyperspectral data analysis for biomarker discovery [58–60].

## Conclusion

The application of hyperspectral imaging in eye research is evolving. Early clinical studies indicate that the technology has transformative potential for biomarker discovery, with scope for the identification of imaging signals of disease-relevant molecules including amyloid β, phosphorylated tau, retinal photopigments, melanin and more. Continued advances in data analysis methods, coupled with the increased availability of affordable and user-friendly cameras will see hyperspectral imaging become integral to ophthalmic practice in future.
